# Student placement and skill ranking predictors for programming classes using class attitude, psychological scales, and code metrics

**DOI:** 10.1186/s41039-018-0075-y

**Published:** 2018-06-28

**Authors:** Ryosuke Ishizue, Kazunori Sakamoto, Hironori Washizaki, Yoshiaki Fukazawa

**Affiliations:** 1 Department of Science and Engineering, Waseda University, Tokyo, Japan; 2 National Institute of Informatics/JST PRESTO, Tokyo, Japan

**Keywords:** Machine learning, Programming class, Placement, Psychological Scale

## Abstract

In some situations, it is necessary to measure personal programming skills. For example, often students must be divided according to skill level and motivation to learn or companies recruiting employees must rank candidates by evaluating programming skills through programming tests, programming contests, etc. This process is burdensome because teachers and recruiters must prepare, implement, and evaluate a placement examination. This paper tries to predict the placement and ranking results of programming contests via machine learning without such an examination. Explanatory variables used for machine learning are classified into three categories: Psychological Scales, Programming Tasks, and Student-answered Questionnaires. The participants are university students enrolled in a Java programming class. One target variable is the placement result based on an examination by a teacher of a class and the ranking results of the programming contest. Our best classification model with a decision tree has an F-measure of 0.912, while our best ranking model with an SVM-rank has an nDCG of 0.962. In both prediction models, the best explanatory variable is from the Programming Task followed in order by Psychological Sale and Student-answered Questionnaire. Our classification model uses 9 explanatory variables, while our ranking model uses 20 explanatory variables. These include all three types of explanatory variables. The source code complexity, which is a source code metrics from Programming Task, shows best performance when the prediction uses only one explanatory variable. Contribution (1), this method can automate some of the teacher’s workload, which may improve educational quality and increase the number of acceptable students in the course. Contribution (2), this paper shows the potential of using difficult-to-formulate information for an evaluation such as a Psychological Scale is demonstrated. These are the contributions and implications of this paper.

## Introduction

Sometimes it is necessary to measure a person’s programming skills^1^. For example, in education, often students must be divided into advanced and intermediate classes based on skill level, motivation to learn, etc. As another example, a company recruiting and placing employees must rank candidates by evaluating programming skills through programming tests, programming contests, etc. However, these processes are burdensome because the evaluator (teacher or recruiter) must prepare, implement, and assess the examination (e.g., placement test or questionnaire regarding class level) to determine programming ability. Moreover, when a teacher conducts such a questionnaire, the interpretation is subjective, which can cause problems in a class with two or more assigned teachers or if the teacher changes. Several other problems may also exist. For example, some students only memorize the answers of past examinations, while other students cram the night before a test.

This paper aims to properly place or rank students using an easier method than the traditional time-consuming examination. We focus on a class for second-year undergraduate students learning to program in Java at Waseda University. In this class, students participate in a programming contest at their department’s orientation about a month after the semester begins. The purpose of the contest is to increase student’s interest in programming. However, the contest is designed to evaluate programming skills. Around the same time as the contest, students are divided into an advanced class and an intermediate class according to the placement examination by the teacher.

In this paper, we try to substitute the examination with a questionnaire, which asks students about their class attitude, and the results of a Programming Task in the class. This information is then used to create a machine-learning model to predict the placement results as well as the ranking results of the programming contest. Three explanatory variables are used: (1) Programming Task, (2) Student-answered Questionnaire, and (3) Psychological Scale. The Programming Task evaluates student’s objective class attitudes and degree of understanding. The Student-answered Questionnaire gages student’s subjective class attitudes and understanding as a self-assessment based on experiences within class hours. The Psychological Scale indicates student’s self-assessment based on experiences outside class hours.

The Psychological Scale affects student’s academic performance Duckworth et al. (2007) and Duckworth and Gross (2014), and Duckworth and Quinn (2009). Previous studies have clearly employed Programming Task and Student-answered Questionnaire in the evaluation. They also demonstrate the relationship of these two variables with the Psychological Scale. However, the Psychological Scale is not used as an evaluation criterion. Thus, this study employs three sets of explanatory variables.

**Fig. 1:**
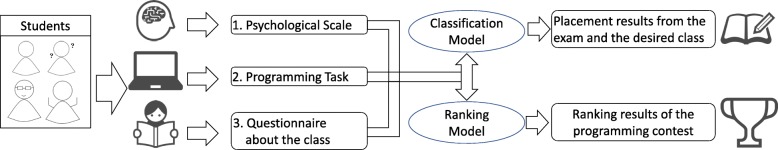
Overview of our two prediction models with explanatory variables and objective variables

Figure 1 shows our two prediction models. The best classification model to predict the placement results, which we created with a decision tree, has a precision of 0.943, recall of 0.908, and an F-measure of 0.912. The best ranking model to predict the ranking results of the programming contest, which we created with an SVM-rank, has an nDCG of 0.962. Additionally, we evaluated the effects of the explanatory variables on the placement results and the programming contest. We investigated 9 factors affecting the placement results and 20 factors affecting the ranking results of the programming contest.

The contributions of this paper are:

- This method can automate some of the teacher’s work, which may improve the quality of the lessons and increase the number of acceptable students.
- The evaluation shows that the model changes the students’ class attitude.

## Related work

Methods to support education are mainly divided into student support and teacher support. Many studies have focused on student support in programming education such as the visualization program execution status (Ishizue et al. 2017b; 2018) and a method to learn a language based on another language already learned (Li et al. 2017). This study focuses on teacher support.

### How are students’ programming skills traditionally assessed?

Traditionally, students’ programming skills are assessed by whether they can solve Programming Tasks. McCracken et al. (2001) surveyed a multi-national, multi-institutional study of assessments of programming skills of first-year CS students. They defined the general evaluation (GE) criteria and the degree of closeness (DoC) evaluation criteria. The GE criteria objectively assess how accurately students implement their solutions. The DoC criteria subjectively evaluate the results of the abstraction and transformation generated sub-problems into sub-solutions.

The GE criteria consist of:

- Execution: Does the program execute without errors? (30 points)
- Verification: Does the program correctly produce answers to the benchmark data set? (60 points)
- Validation: Does the program represent what is asked for in the exercise specifications? (10 points)
- Style (Optional): Does the style of the program conform to local standards? (10 points)

The total number of points is considered to represent importance.

The DoC criteria consist of:

1. Does the program compile and work?
2. Is part or all of the method missing?
3. Are there meaningful comments, stub code, etc.?
4. Does the source code complete little of the program?
5. Does the source code show that the student has no idea about how to approach the problem?

The results of the programming contest are also used to assess programming skills. Trotman and Handley (2008) indicated that programming contests with automated assessments have become popular activities for training of programming skills. Verdú et al. (2012) also indicated that competition is a very important element since the combination of a contest with an automated assessment provides the educational community with an effective and efficient learning tool in the context of teaching programming.

### Can additional variables be used to predict programming skills?

We investigated explanatory variables that can predict general academic skills not only for programming. Prior studies indicate that the Psychological Scale may be an explanatory variable.

We use famous Psychological Scales as explanatory variables in machine learning. The following scales are thought to affect academic performance. Deci and Ryan (1985, 2002) studied intrinsic motivation in human behavior. They defined intrinsic motivation as the life force or energy for an activity and the development of an internal structure. The degree of self-efficacy affects the efficiency of such behavior. According to Bandura (1977), self-efficacy expectancies determine the initial decision to perform a behavior, the effort expended, and the persistence in the face of adversity. Sherer et al. (1982) developed a self-efficacy scale.

The task value is a scale focusing on the value aspect of motivation. According to Eccles and Wigfield (1985), the task value is divided into three subscales (interest value, attainment value, and utility value). Moreover, Ida (2001) further divided attainment value and utility value into two for a total of five subscales. The attainment value is divided into the private attainment value, which is an internal absolute standard that varies by individual, and the public attainment value, which focuses on the superiority/inferiority with others. The utility value is divided into the institutional utility value, which is used when learning is necessary to pass an examination for employment or admission, and practical utility value, which is used when learning is useful in occupational practice. Ida (2001) also proposed a task value evaluation scale.

According to Duckworth and Gross (2014) and Duckworth et al. (2007), and Duckworth and Quinn (2009), self-control is needed to achieve goals that require long-term effort. Self-control allows one to focus on a goal (consistency of interest) and persevere through difficulties (perseverance of effort). They called this combination Grit, and developed an evaluation scale.

Goal orientation is divided into three subscales: mastery orientation, performance approach, and performance avoidance. Elliot and Church (1997) examined their influences and factors.

Ota (2010), Ryckman et al. (1990, 1996), and Smither and Houston (1992) developed a multi-dimensional competitiveness. Multi-dimensional competitiveness is divided into three subscales: instrumental competitiveness, avoidance of competition, and never-give-up attitude. Specific questions based on these scales are shown in Section 3.2.1.

Some studies have investigated these Psychological Scales and learning. For example, Robbins et al. (2004) examined the relationship between psychosocial and study skill factors (PSFs) and college outcomes. They found that the best predictors for grade point average (GPA) are academic self-efficacy and achievement motivation. Shen et al. (2007) investigated the influence of a mastery goal, performance-approach goal, avoidance-approach goal, individual interest, and situational interest on students’ learning of physical education. They reported that a mastery goal is a significant predictor to recognize of situational interest.

We have used class attitude as an explanatory variable for machine learning. Class attitude is also thought to affect the understanding of class content. For example, Saito et al. (2017) studied the relationship between attitudes and understanding of programming with an emphasis on the differences between text-based and visual-based programming.

### How is machine learning previously used in relevant areas?

In this paper, we use classification and ranking machine learning. Various fields, including education, have used machine learning.

Some studies actually predict students’ grades or scores by machine learning. Okubo et al. (2017) studied a method to predict students’ final grades using a recurrent neural network (RNN) and a time series of learning activities logs (e.g., attendance, quiz, and report) in multiple courses. Yasuda et al. (2016) proposed an automatic scoring method for a conversational English test using automatic speech recognition and machine learning techniques.

Some studies use machine learning to find students who need assistance. Ahadi et al. (2015) and Castro-Wunsch et al. (2017) propose methods to automatically identify students in need of assistance. They predict such students using students’ source code snapshot data by machine learning approaches such as decision trees. Hong et al. (2015) implemented a function to the learning system called SQL-Tutor, which identifies students who will abandon the programming task and provides encouragement by displaying motivational messages.

Additional studies have investigated dropouts. Kotsiantis et al. (2003) proposed a prototype web-based support tool using a Naive Bayes algorithm, which can automatically recognize students with a high probability of dropping out. Márquez-Vera et al. (2016) predicted the high school dropout rates of students at different steps in a course to determine the best indicators for dropping out.

It takes time and effort to appropriately categorize students as class size increases. Sohsah et al. (2016) classified educational materials in low-resource languages with machine learning. Machine learning is used not only for teachers but also for school cost problems. Jamison (2017) tried to solve the problem of a declining enrollment rate of students accepted at a given college or university due to academic, economic, and logistical reasons by machine learning.

This paper uses three different kinds of explanatory variables. Such a dataset is called multi-view or multi-source data. Machine learning dealing with this kind of data is called multi-view learning. According to the latest survey of Zhao et al. (2017), multi-view learning has made great advances in recent years. Multi-view learning is machine learning that considers learning from multiple views to improve the general performance. Although this paper uses a traditional method, if this method is applied, our machine learning model may further improve the performance in the future.

## Method

We used supervised machine learning to predict students’ placement results (classification problem) and the ranking results of the programming contest (ranking problem) for a Java programming class at Waseda University. Three sets of explanatory variables were employed: (1) Psychological Test, (2) Programming Task, and (3) Class Questionnaire. Then, we found the best algorithm and the best combination of explanatory variables. The results were used to create and evaluate models for both problems. For the classification problem, we used a Python library called *malss* (https://github.com/canard0328/malss) for machine learning developed by Kamoshida and Sakamoto (2016). For the ranking problem, we used a C language library called SVM^rank^ (www.cs.cornell.edu/people/tj/svm_light/svm_rank.html) for rank learning. The ranking SVM algorithm was developed by Joachims (2002, 2006). The ranking SVM learns by minimizing the error of the order relation when comparing each element with the correct order by making a set of two elements in the sample. We used this program because it is free for scientific use, and we expected the calculation to be fast because the program is written in C language. We published a program to create and evaluate models using these libraries on the following webpages:

- https://github.com/RYOSKATE/CSVFormatter
- https://github.com/RYOSKATE/ProgrammingSkillPredictor

This paper investigated the following research questions (RQs):

- RQ1: How much does each explanatory variable predict the placement results?
- RQ2: How much does each explanatory variable predict the programming contest ranking?
- RQ3: What is the best combination of explanatory variables to predict the placement results?
- RQ4: What is the best combination of explanatory variables to predict the ranking results of the programming contest?

### Participants

This study included 65 second-year undergraduate students at Waseda University (Japan) enrolled in a Java programming class. This class is equivalent to a CS1 level. In this class, students participate in a programming contest at their department’s orientation about a month after the semester begins. The contest is designed to increase students’ interest in programming. Around the same time as the contest, students complete a placement test. Additionally, the students engage in Programming Tasks, answer a Psychological Test, and complete a questionnaire about the class. Then, the students are placed in either an advanced or intermediate course.

Of the participants, 50 students were placed in the advanced course and 15 students were placed in the intermediate course.

### Explanatory variables

Three materials were prepared as explanatory variables in machine learning: (1) Psychological Scale, (2) Programming Task, and (3) Class Questionnaire.

### 1. Psychological Scales

Participants completed a psychological test. Table 1 shows the questions. Each question was evaluated on a seven-level scale: 97) Strongly Agree, (6) Agree, (5) Somewhat Agree, (4) Neutral, (3) Somewhat Disagree, (2) Disagree, and (1) Strongly Disagree.

**Table 1:** Psychological questions

No.	Statements
1	I like programming.
2	I am good at programming.
3	I feel learning to program is interesting.
4	Programming is necessary for my desired job/advancement examination.
5	Programming is useful for desired job/advancement examination.
6	Programming is necessary for practice in my desired occupation.
7	Programming is useful in my desired occupation.
8	I think that learning to program helps me grow as a person.
9	I think that other people respect those who are proficient at programming.
10	I think that to learn programming can be bragging.
11	Setbacks don’t discourage me.
12	I am diligent.
13	I finish whatever I begin.
14	I am a hard worker.
15	I often set a goal but later choose to pursue a different one.
16	I have difficulty maintaining my focus on projects that take more than a few months to complete.
17	New ideas and projects sometimes distract me from previous ones.
18	I am obsessed with a certain idea or project for a short time but later lose interest.
19	I want to learn to improve my abilities.
20	I want to learn new things and increase my knowledge.
21	I want to learn more so that others do not think poorly of me.
22	I want to learn properly so as not to produce bad results.
23	I learn to earn higher test and evaluation results than those around me.
24	When learning something, I like to earn better grades and higher evaluations than other people.
25	By competing, I can enhance my ability.
26	Competition motivates me.
27	If it is boring, I compete with other people to make it interesting.
28	I do not like to compete.
29	I want to avoid competing if possible.
30	I do not want to lose.
31	I feel strongly that I do not want to lose.

Table 2 shows the Psychological Scales corresponding to each question. Question 1 measured intrinsic motivation. Question 2 measured self-efficacy. We used simple statements such as “I like ∼.”, and “I am good at ∼.”. Questions 3 to 10 were based on the task value scale (Eccles and Wigfield 1985). We used question statements developed by Ida (2001). Questions 11 to 18 were based on the Short Grit Scale (Duckworth and Gross 2014; Duckworth and Quinn 2009; Duckworth et al. 2007). We used question statements developed by Nishikawa et al. (2015). Questions 19 to 24 were based on goal orientation (Tanaka and Yamauchi 2000). Questions 25 to 31 were based on multi-dimensional competitiveness (Ota 2010).

**Table 2:** Psychological Scales of each question

No. of question	Psychological scale	Subscale
1	Intrinsic motivation	—
2	Self-efficacy	—
3	Task values	Interest value
4–5	Task values	Institutional utility value
6–7	Task values	Practical utility value
8	Task values	Private attainment value
9–10	Task values	Public attainment value
11–14	Grit	Perseverance of effort
15–18	Grit	Consistency of interest
19–20	Goal orientation	Mastery orientation
21–22	Goal orientation	Performance avoidance
23–24	Goal orientation	Performance approach
25–27	Multi-dimensional competitiveness	Instrumental competitiveness
28–29	Multi-dimensional competitiveness	Avoidance of competition
30–31	Multi-dimensional competitiveness	Never-give-up attitude

### 2. Programming task

Each Programming Task was from the Aizu Online Judge (AOJ). AOJ is the most famous Online Judging System in Japan. AOJ has many programming problems, ranging from simple ones such as “Hello World” to difficult ones such as ACM-ICPC (https://icpc.baylor.edu/) previous problems. When a user submits his or her program source code via the submission form on the AOJ website, the correctness of the program is verified by executing it on the server side. Table 3 lists the IDs and names of the problems used. Additionally, we ranked each problem according to the difficulty by considering the content and the correct answer rate. A larger number indicates a more difficult level. Moreover, we measured the source code metrics, which students submitted to AOJ. To collect their source code, we used Nightmare, which is a high-level browser automation library written in JavaScript. To measure the metrics, we used Checkstyle, which is a static analysis tool for Java. Due to the simple APIs of each library, an automatic measurement program with 100 to 200 LOC can be easily derived. The maximum values determined by Checkstyle’s default were used.

**Table 3:** Problem id, name, and difficulty of Programming Task of AOJ (all problems are from http://judge.u-aizu.ac.jp/onlinejudge/description.jsp?lang=en? id=ProblemID)

Problem ID	Problem name	Difficulty level
10000	Hello World	1
10001	X Cubic	1
10002	Rectangle	1
10009	Circle	2
10010	Simple Calculator	3
10003	Small Large or Equal	1
10004	Sorting Three Numbers	1
10005	Print Many Hello World	1
10006	Print Test Cases	1
10012	Print Rectangle	1
10013	Print a Frame	2
10016	Grading	2
10019	Sum of Numbers	2
10017	How many ways?	3
10021	Finding minimum String	3
10028	Sort I	3
0121	Seven Puzzle	4
0030	Sum of Integers	4
10014	Print a Chessboard	1
ITP1_5_D	Structured Program I	1
10023	Shuffle	2
10020	Counting Characters	2
1129	HanafudaShuffle	3
10031	Search II	3
1160	How Many Islands?	4
10026	Standard Deviation	1
10020	Counting Characters	1
0011	Drawing Lots	1
1147	ICPC Score Totalizer Software	2
1129	Hanafuda Shuffle	2
2102	Rummy	3
1173	The Balance of the World	3
1166	Amazing Mazes	3
1144	Curling 2.0	4
1133	Water Tank	4
1302	Twenty Questions	4

The following metrics were used to detect if the maximum value was exceeded:

(1) Is Solved, (2) LOC, (3) Boolean Expression Complexity, (4) Class Data Abstraction Coupling, (5) Class Fan Out Complexity, (6) Cyclomatic Complexity, (7) Executable Statement Count, (8) Max Len file, (9) Max Len method, (10) Max Line Len, (11) Max Outer Types, (12) Max Param, (13) NCSS Class, (14) NCSS File, (15) NCSS Method, (16) Npath Complexity, and (17) Too Many Methods.

### 3. Questionnaire about the class

We implemented a questionnaire about the class. This questionnaire was created to obtain a subjective evaluation of the students themselves. Participants completed the questionnaire in the class immediately after the placement test. Table 4 shows the questions. All questions were evaluated on a seven-level scale. These questions were created based on the end-of-term questionnaire that Waseda University employs for all classes.

**Table 4:** Questionnaire about the class

No. of question	Questions
1	Are you satisfied with the content of the class so far?
2	How much time do you spend learning class content outside of the class hours per week?
3	Do you try to understand the lesson content?
4	Do you understand the content of this class?
5	Do you think that class materials are easy to understand?
6	Do you think that the contents of the exercises and homework are difficult?
7	Do you think that the number of tasks and the amount of homework are too much?
8	Do you think that teachers grasp the degree of understanding of students when preparing class content?
9	Are you interested in competitive programming like AOJ and contests?
10	Do you think that this lesson is meaningful?

### Objective variables

There were two kinds of objective variables: the placement results and the ranking results of programming contest. We predicted each objective variable using the explanatory variables.

### Placement results

Table 5 shows the examination sentences of the assignment test (programming quiz). The programming quiz took 90 min. The quiz also asked each student about preferred class placement: advanced or intermediate (*Hope Class*). Although the examination result was not used as an explanatory variable for machine learning, it was used by the teacher for class placement. The examination result is used only for sample labeling.

**Table 5:** Examination programming quiz

No.	Examination sentence
1	Create a program that computes the sum of natural numbers from 1 to 100 and outputs it to the display. Do not use mathematical formulas.
2	Create a program that calculates the sum of squares from 1 to 100 and outputs it to the display. Do not use mathematical formulas.
3	Create a program to calculate a sequence of numbers (Fibonacci numbers & F(0)=0, F(1)=1, F(n)=F(n-1)+F(n-2)) where the program terminates when F (n) exceeds 10000.
4	Create a program to calculate a sequence of numbers (Tribonacci number & T(0)=0, T(1)=0, T(2)=1, T(n)=T(n-1)+T(n-2)+T(n-3)). where the program is terminated when T (n) exceeds 10000.
5	Create a program to generate 1000 Java random numbers with natural numbers between 0 and 100. Display their maximum value, minimum value, and average value.
6	Create a program that displays the number of bills (10,000 yen, 5,000 yen, 1 thousand yen) and coins (500 yen, 100 yen, 50 yen, 10 yen, 5 yen, 1 yen) needed to pay the random amount entered on a keyboard. The solution should use the fewest bills or coins possible.
7	Create a game to hit a randomly generated integer between 0 and 999. When the user inputs a value smaller than the correct answer, display “it is smaller than the correct answer”. When the user inputs a value larger than the correct answer, display “it is larger than the correct answer”. If the user does not answer correctly after 10 attempts, display “Game Over”.
8	Please indicate the execution result of the following three programs: e.g. for(char c=’A’; c<=’Z’; c++) System.out.print(c); System.out.print("\n");

### Ranking results of the programming contest

Table 6 shows the description sentences of the programming contest. The contest time was 90 min. Each problem was given a maximum score. When a student solved a problem, his or her score was calculated by the following equation:

**Table 6:** Programming contest problems. All problems are available from https: //github.com/AI-comp/Problems2017 (in Japanese)

No.	Description sentence
1	There are three kinds of menus in a certain oil soba shop: regular bowl, large bowl, and god bowl. The regular bowl is *a* grams, the large bowl is more than *b* grams from the regular bowl, and the god bowl is *c* grams more than the large bowl. Kato-kun ate a regular size bowl, a large size bowl, and a god size bowl. How many grams Kato-kun did eat?
2	Kato-kun is trying to make doujinshi as a hobby. Progressing smoothly, a total of *x* pages have been completed so far. Since the exhibition and sale are close, Kato-kun asked the printing company to bind, but according to a complicated situation, the printing company asked him to make the number of pages a multiple of 4. If *x* is a multiple of 4, the doujinshi is completed. If not a multiple, it is necessary to add a page so that it is a multiple of 4. In addition, Kato-kun does not want to reduce the number of pages of doujinshi because it would be a waste of his time and effort. Since the number of pages already created by Kato-kun is given, output the minimum number of newly created pages for printing.
3	Kato-kun decided to clean his room because it was too dirty. However, Kato-kun is lazy, and he found that the room looks clean if things are on the right side of the room. Thus, he decided to clean his room by moving all items to the right side of the room. The state of the room is given as a one-line character string *S*. String *S* consists of only characters *o*, where *o* indicates the location of items and. indicates a place a location without an item. Output a character string representing the state of the room with all items on the right. For example, if *S* is *o*.*ooo*.., the answer is...*oooo*.
4	World Nokémon Championship (WNCS) is a world competition to determine the Nokémon Master. This tournament consists of a qualifying round and a main battle. Top *N* people in the qualifying round play in the main battle, which is a round-robin tournament. As the result of this round-robin battle is given as a table, display the ranking of the main battle in order from the top. In the win/loss table, *c*_*i*,*j*_ represents the result of the person in *i*th place in qualifying against the person in *j*th place. *o* represents the winning of the person in *i*th place, and *x* represents the losing of the person in *i*th place. Since people cannot compete against themselves, − representing *c*_*i*,*i*_ appears in the table, but a battle does not occur. People with the most wins will be at the top of the ranking. If there is tie for the number of wins, the one with a higher ranking in the qualifying round wins.
5	Character string *S* consists of the letters A, N, P. Output YES if the given string *S* can be generated from the string PPAP using the replacement of P = NP, NO if it is impossible. P = NP replaces P with NP or NP with P. For example, the character string NPPAP can be generated by replacing P at the head of PPAP with NP.
6	Kato-kun, who has a strong appetite but does not exercise, decides to squat *N*! times after eating *N* grams of oiled ramen noodles. However, the intelligent Kato-kun realized that *N*! becomes an explosive number and cannot handle the squats. Thus, he decides to complete only the remainder of *N* divided by 2017 times because this year is 2017. Calculate the number of times Kato-kun squats, when *N* is given. (*a*×*b*) mod *p*=((*a* mod *p*)×*b*) mod *p* for the integers *a*,*b* and *p*.
7	A video game called Nokémon GO catches on with the public. Nokémon GO players move through places called NokéStops to collect and try to collect a lot of Nokémons. One day, Ashe, who is a main protagonist, visited *N* NokéStops. The *i*th of these NokéStops exists at coordinates (*p*_*i*_,*q*_*i*_). In addition, there are *M* Nokémons on the field, and they are at coordinates (*x*_*i*_,*y*_*i*_). While staying at a NokéStop, Ashe can collect all Nokémons within a radius of *L* miles from the NokéStop. Due to social demands, it is forbidden to catch Nokémons while moving. Calculate the maximum number of Nokémons that Ashe can collect on this day.
8	Professionals Pass All Problems (PPAP) is a ritual handed down in the Kingdom of Hylule from the old days. This ritual requires two pens, one apple, and one pineapple. This ceremony has been keeping peace in the kingdom of Hylule at the one shrine maiden. Zerda is the heir of the shrine maiden and practices this ritual every day. The ritual is practiced *x* times to master. In other words, Zerda buys more than 2*x* pens, *x* apples, and *x* pineapples to acquire the PPAP. There are *N* Telly’s shops in the kingdom, and *a*_*i*_ pens, *b*_*i*_ apples, and *c*_*i*_ pineapples are sold in one set. All Telly’s shops sell the set. Calculate the minimum amount required for Zerda to master PPAP. Answer -1 if it is impossible to buy the quantity necessary.

Score = the maximum score of the problem × ((remaining time /contest time) + 1) /2

The ranking order was determined according to the summation of the score. The contest score was not used as an explanatory variable for machine learning. The contest score is used only for sample ranking.

### Algorithm selection

This paper used supervised learning algorithms for the classification problem. Six algorithms were tested to create a better model:

- Support Vector Machine with RBF Kernel (SVM)
- Support Vector Machine with Linear Kernel (SVML)
- Logistic regression (LR)
- Decision tree (DT)
- Random forest (RF)
- k-nearest neighbors (NN)

SVM is a method in which the boundary line is defined as the line that maximizes the sum of the margins up to the sample data closest to the boundary line when determining the boundary line to classify the data. It can be used not only for classification but also regression with an excellent recognition performance. In a two-choice prediction, LR is a logistic curve used to calculate the probability of becoming one sided with a value of 0 to 1. DC is a method to represent a branch process in a tree structure and the branching target data from the top to determine the final class. RF is a method to create multiple decision trees by randomly selecting data from the training data and determining the final class by majority voting of the results predicted by each decision tree. NN is a method to classify a class of multiple data nearest itself by a majority vote. These algorithms are very famous and popular in machine learning as Bishop (2006) summarized the principles, the good and bad hands of these algorithms.

*Malss* supports all of these algorithms. When the user passes data as parameters to *malss*, it tries these algorithms with cross-validation and parameter tuning using a grid search, and outputs a prediction model and a performance report with the F-measure. We used *malss* 1.1.2 with Anaconda 5.0.0 on Microsoft Visual Studio Community 2017 Version 15.5.3. We used values close to the default ones of *malss* as parameters. All details can be confirmed by referring to our published program.

To evaluate the prediction quality of the model, we implemented the *stratified five-fold cross-validation*. First, the validation divided the data set into five pieces so that each label had the same ratio. One piece was used for testing. The remaining four were used for learning. The cross-validation calculated the F-measure with precision and recall using each of the five divided data sets as test data.

There are lots of measurements to classify algorithms (e.g., accuracy, recall, precision, specificity, F-measure, AUC). The F-measure is a well-balanced measurement calculated from recall and precision. This paper used the F-measure as a classification measurement due to the calculation time and its popularity for classification problems. If the primary purpose is to detect failed students, it may be important to focus on other measurements such as specificity. For the ranking problem, we used Support Vector Machine for Ranking by SVM^rank^. We also used the stratified five-fold cross-validation, which calculated the normalized Discounted Cumulated Gain (nDCG) for the ranking problem and verified the five divided datasets.

The nDCG was calculated by the following expression:

${\mathrm {DCG=rel}}_{i}+\sum ^{k}_{i=2}\frac {\text {rel}_{i}}{\text {log}_{2}i}$, $\text {nDCG}=\frac {\mathrm {DCG_{predict}}}{\mathrm {DCG_{ideal}}}$

(rel_*i*_ : relevance of the *i*th element in the ranking, *k* : number of elements)

We used the training data as a test set (a closed test). Moreover, to reduce the deviation of the data, after dividing the data, the cross-validation process was repeated nine times. The median value was subsequently used.

We used *svm_rank_learn* and *svm_rank_classify* included by SVM^rank^ V1.00 on Microsoft Visual Studio Community 2017 Version 15.5.3. We created and evaluated models in brute force using the following parameter ranges, which seem to be sufficient:

- Kernel: LINEAR and RBF
- Rescaling method to use for loss: (1) slack rescaling and (2) margin rescaling
- L-norm to use for slack variables: (1) L1-norm and (2) squared slacks
- C: Trade-off between training error and margin: [1, 10, 100, 1000, 10000, 100000, 1000000]
- Parameter gamma in the RBF kernel: [1, 10, 100, 1000, 10000, 100000, 1000000]

All details can be confirmed by looking at our published program.

### Feature selection

In the psychological test, we converted the answers to the 31 questions into scores (1 to 7 points). Then, we calculated the sum of the scores by 15 subscales.

Next, we measured the metrics for all student-solved tasks. The scores ranked by the metric magnitude were used as explanatory variables for machine learning because the number of explanatory variables was enormous when each metric was used for each problem. Moreover, we added the total number of answers, the number of answers per difficulty level [*Number of Solved Tasks (AOJ)*, and *Difficulty Level 1 to 4 (AOJ)*].

Finally, we tried to create a model that improved the evaluation score. We investigated the influence of each explanatory variable and removed ineffective variables to avoid a high variance. First, we used the explanatory variable with the best F-measure. Then, we added the explanatory variable with the next best F-measure. When there is more than one explanatory variable with the best F-measure, we randomly chose one and proceeded to the next step. This procedure was repeated until all variables were added like greedy algorithm. Finally, we regarded the model with the best F-measure in the procedure as the best model in our method.

## Results and discussion

### RQ1: how much does each explanatory variable predict the placement results?

Table 7 shows the classification results. As expected, the F-measure of Hope Class significant, suggesting that the teacher considers Hope Class in the placement, but it is not the sole factor. The explanatory variables of the measured metrics show high F-measures. In particular, Class Fan Out Complexity shows the highest F-measure. For the Psychological Scales, self-efficacy and interest also shows high F-measures, suggesting that these explanatory variables predict the placement results. However, the other F-measures in the Psychological Scales are not very good. For the task value, the interest values are high, but the others are low. Never-Give-Up Attitude shows the lowest F-measure. Questions about the class (Q1–10) show F-measures that are higher than those of Psychological Scales, but are lower than those of the measured metrics. From the Programming Tasks, using AOJ, Number of Solved Tasks (AOJ) and Difficulty Level 2 (AOJ) predict the placement result to some degree. However, Difficulty Levels 1, 3, and 4 (AOJ) show low F-measures.

**Table 7:** The best F-measure of each explanatory variable, algorithm with the best F-measure, nDCG of each explanatory variable, name of each explanatory variable, and meaning of each explanatory variable

F-measure	Algorithm	nDCG	Explanatory variable name	Meaning
0.669	NN	0.851	Q1	Satisfaction with class
0.669	DT	0.837	Q2	Learning time
0.670	SVM(LK)	*0.855*	Q3	Effort to understand the content
0.793	LR	0.893	Q4	Comprehension of class content
*0.669*	DT	0.893	Q5	Ease of understanding class materials
0.713	SVM(LK)	0.867	Q6	Difficulty of tasks and homework
0.702	LR	0.897	Q7	Amount of exercises and homework
0.669	DT	0.842	Q8	Teacher’s understanding of students’ level
0.756	DT	0.865	Q9	Interest in competitive programming
0.795	DT	*0.871*	Q10	Whether the class is meaningful
0.669	SVM(LK)	0.772	Perseverance of Effort	Long-term efforts to achieve the goals
*0.669*	SVM(LK)	*0.845*	Consistency of Interest	Self-control and ability to focus the goal
*0.681*	DT	0.832	Mastery Orientation	Enhance ability
0.669	SVM(LK)	*0.796*	Performance Avoidance	Superior to others
0.669	SVM(LK)	*0.787*	Performance Approach	Avoid situations where one’s incompetence is obvious
0.719	DT	0.805	Instrumental Competitiveness	Achieve another purpose through competition
0.691	DT	*0.791*	Avoidance of Competition	Avoid competition
0.661	SVM(RBF)	0.799	Never-Give-Up Attitude	Do not want to lose
0.827	SVM(LK)	*0.886*	Interest Value	Gain fulfillment and satisfaction
0.674	SVM(RBF)	*0.854*	Institutional Utility Value	Must pass the exam for employment or admission
*0.669*	SVM(LK)	0.864	Practical Utility Value	Useful for work and study
*0.669*	SVM(LK)	0.863	Private Attainment Value	Improve oneself on an absolute scale
0.669	SVM(LK)	0.777	Public Attainment Value	Improve oneself on a relative scale
0.866	LR	*0.882*	Self-efficacy	Confidence of one’s own ability
*0.669*	SVM(LK)	*0.828*	Intrinsic Motivation	Motivation by curiosity and interest
0.734	LR	*0.933*	AOJ	Total number of questions answered
0.669	SVM(LK)	*0.801*	Difficulty Level 1 (AOJ)	No. of answers for level 1 problem
0.826	LR	0.936	Difficulty Level 2 (AOJ)	No. of answers for level 2 problem
*0.669*	SVM(LK)	*0.927*	Difficulty Level 3 (AOJ)	No. of answers for level 3 problem
*0.669*	SVM(LK)	*0.887*	Difficulty Level 4 (AOJ)	No. of answers for level 4 problem
0.850	SVM(RBF)	0.945	isSolved	Rank of AOJ
0.892	SVM(RBF)	0.946	LOC	lines of code
0.887	SVM(RBF)	0.939	Boolean Expression Complexity	No. of &&, ||, &, | and ^
0.852	SVM(RBF)	*0.947*	Class Data Abstraction Coupling	No. of instantiations of other classes
*0.911*	SVM(RBF)	*0.937*	Class Fan Out Complexity	No. of other classes a given class relies on
0.882	SVM(RBF)	0.934	Cyclomatic Complexity	Min no. of possible paths in through source
0.878	SVM(RBF)	0.936	Executable Statement Count	No. of executable statements
0.867	SVM(RBF)	0.935	Max Len file	No. of files exceeding the max LOC (2000)
0.864	SVM(RBF)	0.939	Max Len method	No. of methods exceeding the max LOC (150)
0.852	DT	0.939	Max Line Len	No. of lines exceeding the max characters (80)
0.883	SVM(RBF)	0.943	Max Outer Types	No. of types declared at the outer (o r root) level in a file (1)
0.863	SVM(RBF)	0.946	Max Param	No. of parameters exceeding max (7)
0.880	SVM(RBF)	*0.929*	NCSS Class	No. of classes exceeding the max non-comment lines in the class (1500)
0.881	SVM(RBF)	0.945	NCSS File	No. of files exceeding the max commenting lines in a file including all top level and nested classes (2000)
0.897	SVM(RBF)	*0.931*	NCSS Method	No. of methods exceeding the max non-comment lines in the class (50)
0.894	SVM(RBF)	*0.942*	Npath Complexity	No. of possible execution paths through a function (method)
0.876	NN	*0.943*	Too Many Methods	No. of methods exceeding the max methods at all scope levels (100)
0.841	LR	0.842	Hope Class	Class which each student wants to be in.

*Q*corresponds to numbers of Table 4: Questions in the questionnaire about the class. Numbers in parenthesis indicate the default maximum values

### RQ2: how much does each explanatory variable predict the programming contest ranking?

Table 7 also shows the nDCG as the ranking results. The rankings show similar tendencies as the classification results. Questions about the class (Q1–10) and Psychological Scales are not very good. As expected, the number of answered AOJ questions seems to be related to the score because the problem of AOJ is similar to the problem presented in the programming contest. The explanatory variables of the measured metrics also show high nDCG. These results show that the score of the programming contest is not related to the Psychological Scales or class attitudes, but it is related to the quality of the written source code, which can be measured by the source code metrics.

Additionally, Table 8 shows the medians, variances, and *p* values of each explanatory variables. For example, in the first line, MEDIAN_A means the median value of answers for Q1 in the advanced class, MEDIAN_I means the median value of answers for Q1 in the intermediate class, and MEDIAN means the median value of answers for Q1 from all students. We used Wilcoxon’s signed rank test to calculate the *p* values, which represent the statistically significant difference between the intermediate class and the advanced class. According to this result, the advanced class students’ scores are better than the intermediate class students’ scores. For example, in the questionnaire about class attitudes (Q1–Q10), many students in the advanced class chose more options that mean “Agree” compared to intermediate class. About half of the explanatory variables of Psychological Scales and questionnaire about class attitudes show significant differences. In contrast, all metrics show significant differences. These results show that the advanced class students wrote higher quality codes (e.g., smaller LOC and lower complexity) than the intermediate class students. In particular, no intermediate class students solved the problem of Difficulty Levels 3 and 4 (AOJ).

**Table 8:** Median, variance, and *p* value (between the intermediate class and the advanced class) for each explanatory variable in the intermediate class, advanced class, and overall

Explanatory variable name	All students		Intermediate class		Advanced class		*p* value
	Median	Var	Median	Var	Median	Var	
Q1	5	1.2811	4	1.1484	5	1.165	< 0.01
Q2	4	1.214	4	0.8352	5	1.3359	n.s.
Q3	6	1.8699	5.5	1.8242	6	1.9167	n.s.
Q4	5	2.512	3	1.9176	5	1.79	< 0.001
Q5	5	1.894	4	1.3242	5	1.7645	< 0.01
Q6	5	2.0036	6	1.0549	5	1.9507	< 0.01
Q7	5	1.9816	6	0.9945	5	1.8206	< 0.001
Q8	4	1.8807	3.5	1.8077	5	1.8138	0.1
Q9	5	2.2396	4	4.1319	5	1.5561	n.s.
Q10	6	1.34	4.5	2.4176	6	0.8206	< 0.05
Perseverance of Effort	19	20.6825	18.5	30.2692	19	18.2466	n.s.
Consistency of Interest	16	18.9263	15.5	12.7473	16	20.9728	n.s.
Mastery Orientation	12	3.171	11	6.533	12	2.1395	n.s.
Performance Avoidance	11	4.6452	11	5.456	11	4.5204	n.s.
Performance Approach	10	6.36	10	5.456	10	6.7083	n.s.
Instrumental Competitiveness	14	15.1567	13	14.5549	14	15.0825	n.s.
Avoidance of Competition	8	8.2401	7.5	9.9176	8	7.949	n.s.
Never-Give-Up Attitude	10	7.3692	9.5	8.5549	11	6.9209	n.s.
Interest Value	6	1.3292	5	2.2637	6	0.4991	< 0.001
Institutional Utility Value	11	5.3364	10.5	6.1538	12	4.8146	< 0.1
Practical Utility Value	12	4.8438	10.5	6.0275	12	4.1412	< 0.05
Private Attainment Value	4	2.3369	4	2.4396	5	2.2066	< 0.1
Public Attainment Value	10	7.8761	11	5.8022	10	8.375	n.s.
Self-efficacy	4	2.6288	1.5	2.5275	5	1.4396	< 0.001
Intrinsic Motivation	4	2.2202	4.5	2.1319	4	2.2789	n.s.
AOJ	17	52.0312	14	20.9945	18	46.517	< 0.001
Difficulty Level 1 (AOJ)	13	6.1429	13	13.9396	13	3.8019	n.s.
Difficulty Level 2 (AOJ)	4	8.085	1	1.6044	5	6.5978	< 0.001
Difficulty Level 3 (AOJ)	0	7.6912	0	0	1	8.5051	< 0.001
Difficulty Level 4 (AOJ)	0	1.53	0	0	0	1.9218	n.s.
isSolved	79.29	344.16	82.51	37.6	74.79	406.57	< 0.05
LOC	74.71	350.43	79.49	46.1	70.74	415.93	< 0.05
Boolean Expression Complexity	74.82	361.24	78.43	52.8	71.32	429.5	< 0.05
Class Data Abstraction Coupling	76.38	327.24	80.09	44	73.18	390.86	< 0.05
Class Fan Out Complexity	78.71	318.34	81.54	35.22	72.44	378.92	< 0.05
Cyclomatic Complexity	77.68	353.01	81.38	38.52	71	417.03	< 0.05
Executable Statement Count	75.65	358.41	80.66	41.81	70.32	422.71	< 0.05
Max Len File	75.91	334.89	78.84	49.28	71.15	397.93	< 0.05
Max Len Method	77.53	312.96	80.97	35.52	74.32	372.57	< 0.05
Max Line Len	79.88	296.87	82.03	27.47	77.03	351.78	< 0.05
Max Outer Types	81.97	318.33	84.16	24.24	76.71	373.56	< 0.05
Max Param	80.82	316.14	85.24	23.76	76.76	369.61	< 0.05
NCSS Class	79.03	315.06	83.22	31.4	76.35	371.67	< 0.05
NCSS File	78.65	328.85	81.21	37.45	73.32	388.76	< 0.05
NCSS Method	79.82	283.99	82.12	31.88	76.47	338.67	< 0.05
Npath Complexity	80.32	303.62	83.68	28.37	77.12	360.66	< 0.05
Too Many Methods	81.26	301.9	84.53	22.08	77.15	357.36	< 0.05
Hope Class	1	0.1083	1.75	0.2253	1	0.01	< 0.001

These results indicate that higher level students can be identified as they can solve such problems and should be into advanced class when combined with other explanatory variables. However, this explanatory variable alone cannot predict the placement result accurately.

### RQ3: what is the best combination of the explanatory variables to predict the placement results?

We added explanatory variables one-by-one like a greedy algorithm. The best F-measure has a value of 0.912 (recall is 0.908, precision is 0.943, and specificity is 0.933) with DC using the following nine explanatory variables: (1) Q5 about the ease of understanding class materials, (2) Consistency of Interest, (3) Mastery Orientation, (4) Practical utility value, (5) Private Attainment Value, (6) Intrinsic Motivation, (7) Difficulty Level 3 (AOJ), (8) Difficulty Level 4 (AOJ), and (9) Class Fan Out Complexity. The F-measures of these explanatory variables are in bold in Table 7. Adding more explanatory variables actually decreases the F-measure. Table 9 shows the F-measure of each algorithm and the best model. These results show that DC is the best algorithm.

**Table 9:** F-measure of each algorithm for the best score (five-fold nested cross-validation)

Algorithm	F-measure
Support Vector Machine (RBF Kernel)	0.911528
Random Forest	0.882026
Support Vector Machine (Linear Kernel)	0.885752
Logistic Regression	0.872234
Decision Tree	0.912308
k-Nearest Neighbors	0.909317

**Fig. 2:**
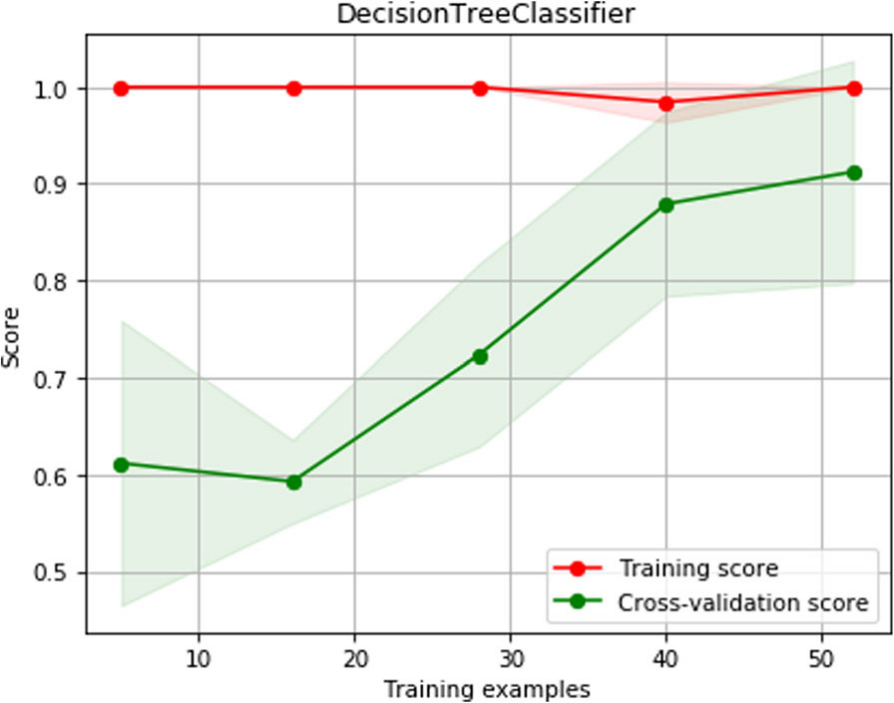
Learning curve of DC with an F-measure of 0.912

Figure 2 shows the learning curve of DC. Improvement in the cross-validation score accompanied by an increase in the learning data is not saturated (continues to improve), indicating a high variance (over-fitting). Thus, employing more training samples can reduce the effect of over-fitting, leading to improvements in the high variance estimator.

RQ1 implies that the results should contain many explanatory variables based on the measured metrics. However, we did not expect questions about class attitudes, Psychological Scales, and Difficulty Levels 3 and 4 (AOJ) to be included as explanatory variables because they showed low F-measures in the previous section. It is thought that these variables perform by combining with the former explanatory variables.

### RQ4: what is the best combination of explanatory variables to predict the programming contest ranking?

Similar to RQ3, we added explanatory variables one-by-one like a greedy algorithm. The best nDCG has a value of 0.962 with DC using the following 20 explanatory variables: (1) Q3 about effort to understand the contents, (2) Q10 about whether the class is meaningful, (3) Consistency of Interest, (4) Performance Avoidance, (5) Performance Approach, (6) Avoidance of Competition, (7) Interest Value, (8) Institutional Utility Value, (9) Self-efficacy, (10) Intrinsic Motivation, (11) Total number of answered questions of AOJ, (12) Difficulty Level 1 (AOJ), (13) Difficulty Level 3 (AOJ), (14) Difficulty Level 4 (AOJ), (15) Class Data Abstraction Coupling, (16) Class Fan Out Complexity, (17) NCSS Class, (18) NCSS Method, (19) Npath Complexity, and (20) Too Many Methods. The nDCGs of these explanatory variables are in bold in Table 7. Adding more explanatory variables actually decreases the nDCG.

RQ2 implies that the results should contain many explanatory variables based on the measured metrics. However, some explanatory variables, which show a low nDCG in the previous section, are included as an element of this combination. It is thought that these variables perform by combining with the former explanatory variables. Therefore, Psychological Scales and the questionnaire on class attitude are also effective in combination with metrics.

In particular, the explanatory variables used for both models such as Consistency of Interest, Intrinsic Motivation, Difficulty Levels 3 and 4 (AOJ), and Class Fan Out Complexity, are considered to have strong relationships with the results.

### Threats to validity

The questionnaires were conducted after the placement test. This could affect the results. Moreover, the best combination may be a local solution. These are threats to the internal validity.

These results are from one class. If this experiment is repeated with another group or organization, the results may differ. Furthermore, the amount of data is small. These are threats to the external validity.

### Conclusion

Machine learning is used to predict both the placement results without a traditional placement examination and the programming skill level without a programming contest. The explanatory variables are Psychological Scales, Programming Tasks, and Student-answered Questionnaires. The target variable is the placement result based on an examination facilitated by a teacher. We investigated how the above three sets of explanatory variables affect the results. Finally, we created a classification model with a precision, recall, and F-measure of 0.912 and a ranking model with nDCG of 0.96172.

If teachers use our method, they can automate evaluations, which may reduce their workload, enhance the education quality, and positively impact students’ class attitude. These are the major contributions and implications of this paper.

However, this research has some limitations. Although our method should be applicable when using the same kinds of variables, its behavior when applying it to other datasets has yet to be confirmed. Our model exhibits a good performance. Because its recall and specificity are not 100%, how to use and operate this model in the field of actual education remains debatable. For example, we need to think about follow-up when the predictor mistakenly classifies a student. Additional improvements may be possible. For example, a superior algorithm compared to those in this study may exist. Regardless of these limitations, our method can be expanded to include other situations such as companies’ recruitment and placement.

The novelty of our method is that it adds the Psychological Scale to traditional evaluation criteria. Our study enables automatic placement based on a multifaceted evaluation using difficult-to-formulate information. This paper demonstrates the feasibility of evaluations using explanatory variables such as the Psychological Scale, which could not be previously employed in machine learning, and suggests that it may be possible to automate education evaluations. In the future, we plan to improve the prediction performance of our method by enhancing the algorithms and adding other explanatory variables.

## Abbreviations

GE: General evaluation
DoC: Degree of closeness
PSFs: Psychosocial and study skill factors
GPA: Grade point average
RNN: Recurrent neural network
RQs: Research questions
AOJ: Aizu Online Judge
SVM: Support Vector Machine with RBF Kernel
SVML: Support Vector Machine with Linear Kernel
LR: Logistic regression
DT: Decision tree
RF: Random forest
NN: k-nearest neighbors
NDCG: Normalized discounted cumulated gain

## Footnotes

1 This paper is an extended version of a paper “Student Placement Predictor for Programming. Class Using Classes Attitude, Psychological Scale, and Code Metrics.” presented at the 25th International Conference on Computers in Education (ICCE 2017). In the previous paper, we only predicted the student placement result. In this paper, we try to predict student skill ranking using programming contest, add new algorithm for classification. In summary, this paper demonstrates the applicability of our method to real programming class.
